# Changes in the Networks of Attention across the Lifespan: A Graphical Meta-Analysis

**DOI:** 10.3390/jintelligence12020019

**Published:** 2024-02-10

**Authors:** Raymond M. Klein, Samantha R. Good, John J. Christie

**Affiliations:** Department of Psychology & Neuroscience, Dalhousie University, Halifax, NS B3H 4R2, Canada; samgood@dal.ca (S.R.G.); john.christie@dal.ca (J.J.C.)

**Keywords:** alerting, orienting, executive control, attention, development, lifespan, graphical meta-analysis

## Abstract

Three Posnerian networks of attention (alerting, orienting, and executive control) have been distinguished on the bases of behavioural, neuropsychological, and neuroscientific evidence. Here, we examined the trajectories of these networks throughout the human lifespan using the various Attention Network Tests (ANTs), which were specifically developed to measure the efficacy of these networks. The ANT Database was used to identify relevant research, resulting in the inclusion of 36 publications. We conducted a graphical meta-analysis using network scores from each study, based on reaction time plotted as a function of age group. Evaluation of attentional networks from childhood to early adulthood suggests that the alerting network develops relatively quickly, and reaches near-adult level by the age of 12. The developmental pattern of the orienting network seems to depend on the information value of the spatial cues. Executive control network scores show a consistent decrease (improvement) with age in childhood. During adulthood (ages 19–75), changes in alerting depend on the modality of the warning signal, while a moderate increase in orienting scores was seen with increasing age. Whereas executive control scores, as measured in reaction time, increase (deterioration) from young adulthood into later adulthood an opposite trend is seen when scores are based on error rates.

“For our discipline, for understanding the organization (and disorganization) of behaviour and thought by our mind and brain (cf. Hebb 1949), the concept of attention is of fundamental importance. This is not because, as James (1890) famously said, “everyone knows what attention is”; but rather because everyone’s behaviours and thoughts are mediated, in one way or another, by some aspect of attention”.

(Klein 2022)

## 1. Introduction

A little over a century ago, Titchener (1908) described attention as “the nerve of the whole psychological system”. Despite this early placement of the concept at the crux of psychological thinking, attention, along with other mental constructs, was temporarily neglected in the first half of the 20th century because of the rise of behaviourism, which cast such mentalisms into disrepute. Watson’s so-called behaviourist manifesto (Watson 1913), for example, asserted that psychology “need no longer delude itself into thinking that it is making mental states the object of observation”.

The neglect began to wane in the middle of the last century, as it was realized that mental constructs were useful, if not necessary. There were several contributing factors (for reviews, see (Klein 2022) and (Lachman et al. 1979)), including the following: accidents during WW2 initially attributed to human failure were recognized as due to limitations in human information processing that had not been anticipated in the design of the machines of war; the field of cybernetics was born and a metric for measuring information and its transmission through a limited capacity channel was developed (Shannon 1948); the value of mental models was proposed (Craik 1943); even animal learning researchers recognized the need for “cognitive maps” (Tolman 1948); Hebb’s landmark book (Hebb 1949), *The Organization of Behavior: A Neuropsychological Theory*, and his other works, laid the foundation for a cognitive revolution; later, Chomsky (1959) would demolish Skinner’s attempt to understand verbal behaviour in Watsonian terms.

The need for a revival of interest in attention was highlighted early in Hebb’s book, “Since everyone knows that attention and set exist, we had better get the skeleton out of the closet and see what we can do with it. (Hebb 1949, pp. 4–5)”. The empirical and theoretical work Hebb was hoping for was begun in earnest by Broadbent (1958) and others in the second half of the 20th century. A recent four-volume compendium entitled *The Psychology of Attention*, assembled by Michael Posner (2016), clearly demonstrates that attention is no longer “in the closet”. This multi-volume set presents the following topics: the history of humankind’s interest in attention; landmark empirical and theoretical works on the topic; neuroscientific studies that have revealed how attentional functions are represented in and implemented by the brain; and applied research showing how attention matters in everyday modern life.

In this article, we will begin by summarizing Posner’s taxonomy, which organizes different aspects of attention, and by briefly describing the attention network tests that were developed to measure the efficacy of these different aspects. This will lay the foundation for our main goal: to see what the literature using these tests has revealed about how the networks of attention proposed by Posner might change across the lifespan.

## 2. Networks of Attention

Developed by Michael Posner and his collaborators (Fan et al. 2002), the Attention Network Test (ANT) was based on Posner’s behavioural (Posner and Boies 1971) analysis of the components of attention and his later neuroscientific analysis of the networks (Posner and Petersen 1990) that mediate alertness, orienting, and executive control.

Alertness is about the achievement and maintenance of a readiness to perceive and respond to stimuli. Although alertness can entail relatively slow (tonic) changes in mental state (such as those related to the sleep–wakefulness cycle or fatigue), the original ANT focussed on rapid changes initiated by environmental signals that cause a change in arousal and/or might signal an impending and important event; hence, readiness to process and respond. Orienting is about the focussing of attention on one particular input pathway. Within the visual modality, this is typically about spatial locations; but, we can allocate our attention to different modalities or to different attributes within a modality. Whereas spatial orienting can be either overt (accomplished by eye movements) or covert (shifts of attention without a shift in gaze direction), typically, the ANTs are designed to explore covert orienting. Orienting can be controlled by both bottom–up (reflexive, or exogenous) and/or by top–down (voluntary, or endogenous) mechanisms, a difference that is also reflected in some ANT variants. Executive control refers to a complex set of cognitive functions that include planning, error detection, and conflict resolution. For the purpose of assessing executive control, all ANTs use a variant of the “flanker” effect (Eriksen and Eriksen 1974), in which participants respond to targets defined by their location, while nearby items might be different from (incongruent) or the same as (congruent) the target stimulus. In light of the theme of this special issue, we will examine lifespan changes in attention, as revealed by the use of any of the ANTs, that is, tests developed for measuring the efficacy of the networks of attention.

## 3. The ANTs

**Figure 1:**
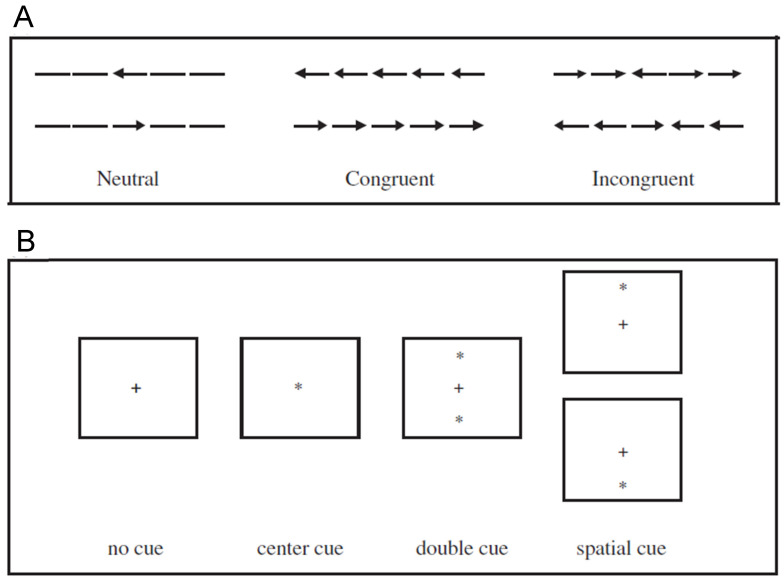
Possible target arrays (**A**) and cue conditions (**B**) used in the ANT-O. In (**A**) the participant’s task is to report the direction of the central stimulus. In (**B**) the + is the fixation stimulus.

When performing the original ANT (hereafter ANT-O), participants are tasked to indicate, with a speeded button press response, the direction of an arrow target, presented either above or below fixation. To explore the participant’s ability to deal with conflicting information (an executive control function) the target arrow might be surrounded by incongruent or congruent arrows or neutral stimuli (see Figure 1A). Executive control is assessed using the deleterious effect of incongruent versus congruent distractors. The target-containing arrays are preceded by four different cue conditions (see Figure 1B): no cue, central cue, double cue and spatial cue. Alertness is assessed by comparing the no cue condition with the double cue condition. Following a spatial cue, the target appears with 100% probability at the location of the cue. Orienting is assessed by comparing the central cue condition with the spatial cue condition. As noted by Klein (2003), the ANT-O is a prime example of the application of mental chronometry (Donders [1868] 1969; Posner 1978) to the assessment of attention, with each subtraction score (see Table 1) providing information about the efficacy of each network of attention. Most scholars who have used an ANT have consequently focussed on reaction times. That noted, and setting a model for others, Fan et al. (2002) were careful to report both RT and accuracy for each of the 12 conditions of the ANT. Unfortunately, many have not followed this model. It is also noteworthy that the original ANT was designed to keep the network scores mathematically independent. In a condensed version, often used in neuroimaging studies, the double cue condition is dropped, and the central cue condition figures in two subtractions (Alerting = none − central; Orienting = central − spatial), thereby sacrificing this independence.

**Table 1:** Subtractions (using reaction time or error rates) that generate network scores.

Network	Subtraction: ANT; ANT-C	Subtraction: ANT-I
Alerting	no cue − double cue	no tone − tone
Orienting	central cue − spatial cue	invalid cue − valid cue
Executive	incongruent − congruent	incongruent − congruent

One variant of the ANT was developed by Rueda et al. (2004), specifically for use with children, for whom a more engaging version was deemed essential (cf. Berger et al. 2000). The child ANT (hereafter, ANT-C) was modelled closely on the original ANT-O. The arrows were replaced with colourful fish, the cue conditions remained the same, and feedback (visual and auditory) was added following correct and incorrect responses. The subtraction scores remained the same as for the ANT-O.

The ANT-I was developed by Callejas et al. (2004), with the “I” signifying the intention to be able to measure the interaction between orienting and alerting, which, by design, is not possible with the ANT. The ANT-I differs from the original ANT-O in two important respects: the spatial cues are uninformative about where the target might appear, and alerting is generated by an auditory warning signal (which is present on half of all trials).

The difference in how orienting is assessed using the ANT-O (and ANT-C), as compared to the ANT-I, is noteworthy. By using an informative (100% valid) peripheral cue, the original ANT allows for the possibility that the orienting score reflects an unknown combination of capture of attention by the peripheral cue with volitional allocation of attention to the location indicated by the cue. Using the terminology first introduced by Posner (1980), the ANT-O potentially confounds exogenous with endogenous control of orienting. As noted by Klein (2009), there is considerable evidence that these two forms of orienting are quite different, not only in their mode of control, but also in terms of the effects upon subsequent processing that they engender. One positive feature of the ANT-I is that the measure of orienting is purely about the exogenous form.

Two modified versions of the ANT-I deserve mention because they feature prominently in the development section. Mullane et al. (2014)^1^ modified the adult ANT-I to provide a second measure of executive control. As described above, all of the ANTs generate an executive control score that is based on the ability of participants to ignore irrelevant items close to the target (the well-known flanker effect). Mullane et al.’s second measure of executive control was provided by the Simon effect (Note: Some refer to this particular example of the Simon effect as “spatial Stroop” because the target itself is spatial), that is, on some trials, a single arrow pointing left or right was presented alone to the left or right of fixation. Because the task is to report the direction of the arrow, the task-irrelevant location should be ignored; how effectively it is ignored is the second measure of executive control. Giovannoli et al. (2021) used the ANTI-V (developed by Roca et al. 2011). As in the ANT-I, the visual cues were uninformative and alertness was generated using an auditory tone. Unlike most ANT-related studies, the targets and distractors were not arrows, but were cars facing either left or right. And, as described in Roca et al. (2011), the ANTI-V generates a measure of vigilance through the inclusion of occasional trials calling for the detection of a slight displacement of the target car.^2^

There are many other tests designed to assess the networks of attention that evolved from the ANT-O (a full review of the various ANTs can be found in Almeida et al. 2021). For present purposes what we have presented above ought to be sufficient, and any minor departures from the three prototypical tests (ANT-O, ANT-C, and ANT-I) that were used in the studies we consider below will be noted as appropriate.

## 4. Lifespan

The value of considering lifespan changes in psychological functions is well recognized (e.g., Baltes et al. 1980), as has been the more specific interest in lifespan changes in attention (e.g., Enns 1990; Plude et al. 1994). Not surprisingly, the components of attention are thought to have different developmental trajectories throughout the human lifespan. Indeed, there are solid lifespan studies of many, individually explored, aspects of attention. It is not our intention to provide a thorough review of this literature, but in light of the emphasis on Posner’s taxonomy, we will simply point out a few relevant examples of lifespan studies each with a distinct area of focus: executive function (e.g., Erb et al. 2023; Zelazo et al. 2004); sustained attention (e.g., Fortenbaugh et al. 2015); and orienting (e.g., via visual search, Hommel et al. 2004; Trick and Enns 1998). There are also some studies that conducted cross-sectional lifespan explorations of multiple aspects of attention in the same sample (e.g., McAvinue et al. 2012; Waszak et al. 2010).

Our focus is on the use of the ANTs to explore changes in the networks of attention across the lifespan. This focus has several advantages, including the following: (1) there will be simultaneous assessment of three networks of attention from the same individuals; (2) methodological homogeneity makes it possible to combine different studies that used the same (or similar) versions of the ANT across similar and different ages; (3) there is a relatively large number of studies that varied age, using otherwise identical methods. For developing a true account of changes in the networks of attention across the lifespan, there are some related disadvantages. Whereas there are versions of the ANT that were developed specifically for children, there are no efforts we are aware of in which these were used to test children younger than 3 years of age, and, for reasons described later, only data from children ~5 years of age and older figured into our analysis. There are several true developmental studies in which a relatively broad range of ages were tested. Most of these studies used the ANT-C. In contrast, while there are many studies of the effects of age upon attention in adults, most of these studies used only two age groups: young and old. Additionally, no studies comparing these age groups have used the ANT-C, which makes the generation of network scores as a continuous function of age difficult.

## 5. Methods

The studies used in the graphic meta-analysis presented below were found in the ANT Database (https://www.attentionnetwork.ca/), or added to it when we learned about them. This database was described in Arora et al. (2020), in which two applications of the database are presented. It is noteworthy that the generation and upkeep of this database subscribe to the PRISMA (Preferred Reporting Items for Systematic Reviews and Meta-Analyses) guidelines. With a few exceptions in the development section, we emphasize studies that included more than one age group. A few retrieved studies were excluded if they did not present network scores for the three networks (or did not present data from which such scores could be generated). Because many studies did not report data about error rates, our focus will be on reaction time; nevertheless, accuracy/errors will be addressed in the general discussion. 

**Figure 2:**
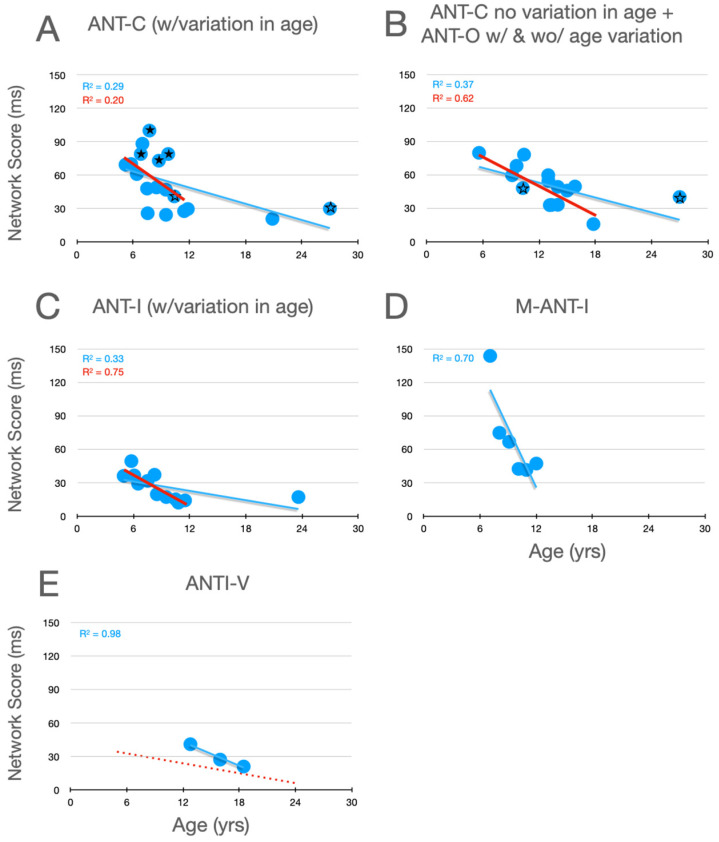
Alertness scores as a function of age in studies that included children. (**A**) Studies of the ANT-C in which age varied. (**B**) Studies of the ANT-C with only one age, and studies of the ANT-O with age varied or not. The stars in B and C are from Rueda et al. (filled stars from experiment 1; unfilled stars from Experiment 2). (**C**) Studies of the ANT-I all with age varied. (**D**) Mullane et al. (2014). (**E**) Giovannoli et al. (2021). In each graph, a best fitting linear function (in blue) is fit to all the data points. In (**E**), the line from panel **C** is reproduced (dotted and red). In (**A**–**C**), a best fitting line (in red) was fit after excluding the young adult participants (>18).

**Figure 3:**
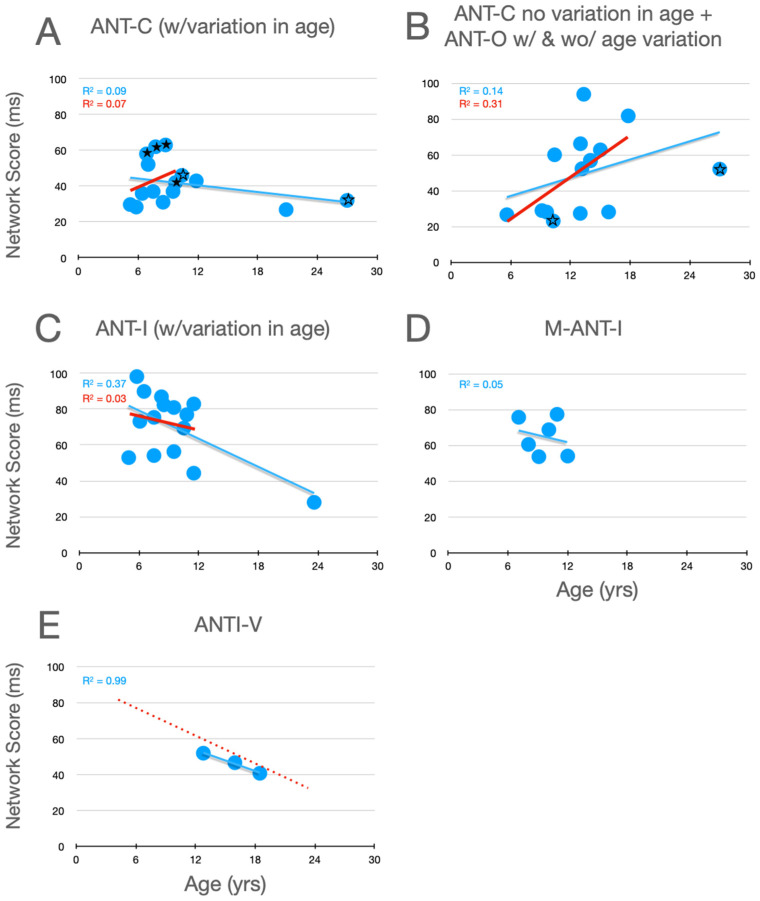
Orienting scores as a function of age in studies that included children. See the caption of Figure 2 for further details.

**Figure 4:**
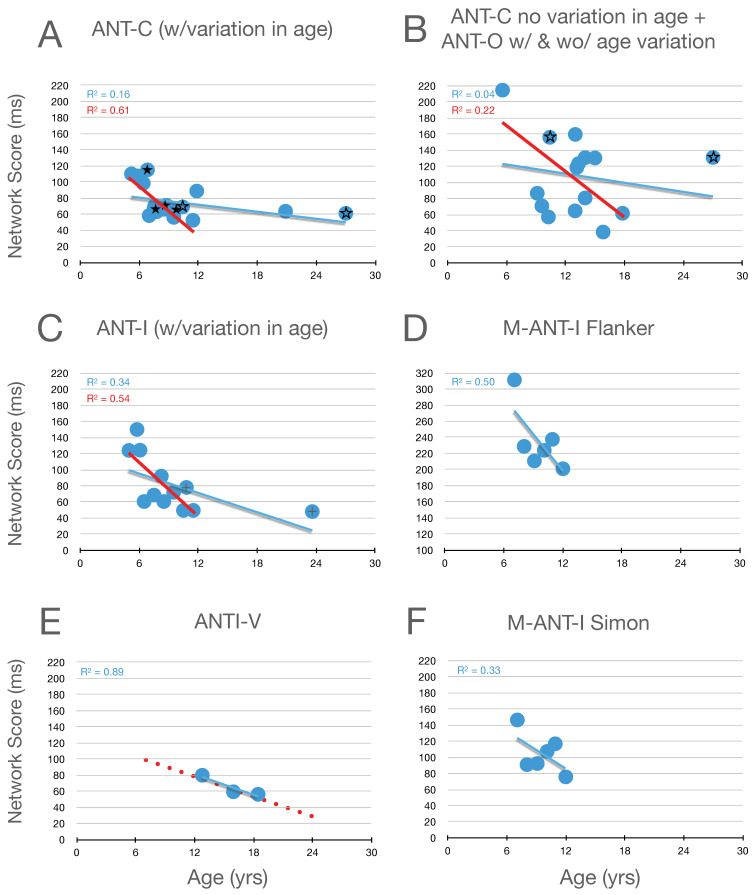
Executive control scores as a function of age in studies that included children. Highlighted data in panel (**C**) are described in the text. In panel (**D**) the scaling is the same as the other panels while the range covered is higher. In panel (**E**) the red dotted line reproduces the linear function fit to all the data in panel (**C**). Data from the Simon effect are presented in panel (**F**). See the caption from Figure 2 for further details.

**Figure 5:**
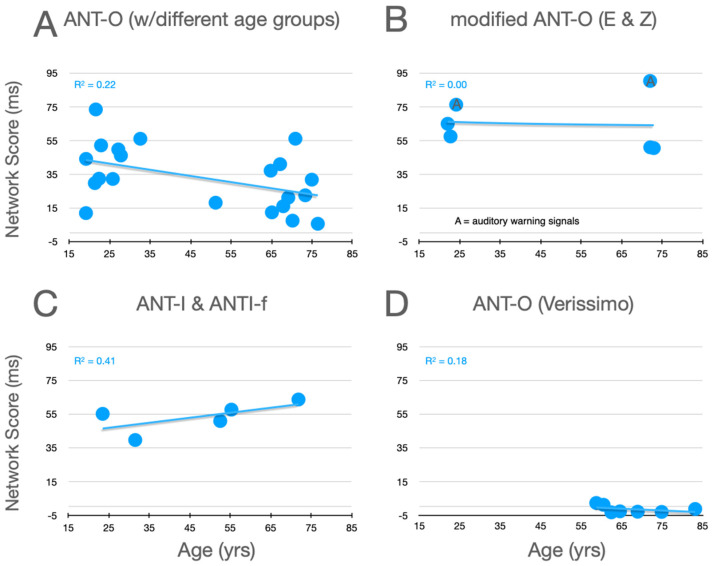
Alertness scores as a function of age from studies that included older adults. (**A**) Ten studies using the ANT-O with two or more groups, one of which included young adults; (**B**) Data from the three conditions tested in the studies by Erel et al. (2020) and Zivony et al. (2020); (**C**) Data from Casagrande et al. (2021). (**D**) Data from Veríssimo et al. (2022). See text for further explanation.

**Figure 6:**
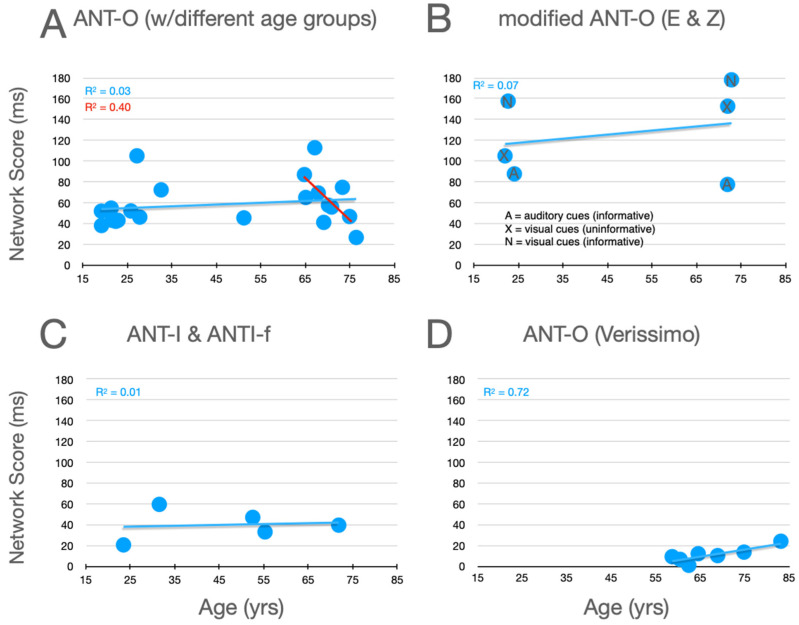
Orienting scores as a function of age from studies that included older adults. See the caption of Figure 5 for further details.

**Figure 7:**
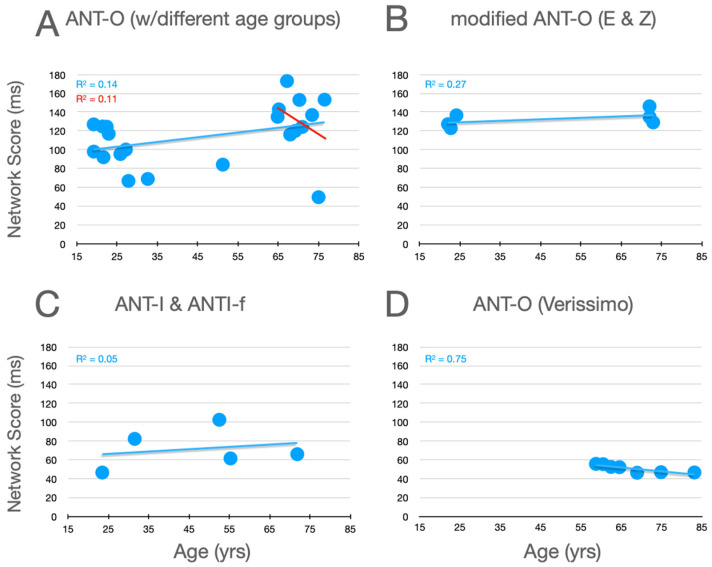
Executive control scores as a function of age from studies that included older adults. See the caption of Figure 5 for further details.

In our graphical analysis, each data point represents the network score (based on reaction time) for a particular age group from a particular study/experiment. Although the number of participants per group (as implied by the numbers reported in Table 2 and Table 3) varies substantially between studies, for present purposes, we have treated each data point equally. There are several reasons for this approach. Because different studies sampled different age groups, it seems appropriate to treat each study as providing “best” estimates for each of the age groups they tested. Relatedly, it seems inappropriate to give some ages more weight than others simply because they were sampled more densely by a researcher than other ages were by other researchers. Finally, even despite the homogeneity associated with the use of similar tests that are presented together (see separate panels in Figure 2, Figure 3, Figure 4, Figure 5, Figure 6 and Figure 7), there are differences that encourage us to treat each sample equivalently. Primarily for descriptive purposes, we have chosen to plot ordinary least squares linear functions (along with R^2^) to the data presented in each data panel. We have little certainty that these are truly linear functions, due to the many holes in the age ranges presented. Readers may imagine, correctly, that some of these patterns might be better fit by other functions (e.g., power, polynomial). We have presented them as linear because they are the simplest and make potential alternative functions easier to see than points without a descriptive function.

## 6. Measuring the Networks of Attention during Childhood

The development of attention during childhood is a topic of great interest and importance. The ability of the ANTs to simultaneously provide indices of the efficacy of three components of attention makes it an appealing tool for exploring the development of attention. However, the ANT was designed for adults and, as suggested by Berger et al. (2000), children will provide more robust and reliable data when they are engaged. Mindful of this advice, Rueda et al. (2004) developed a child-friendly version, the ANT-C (described briefly above), and used it to conduct the first developmental study.

Since its development, the ANT-C has been widely used in studies with children and, occasionally, for comparison purposes, young adults have been tested using the ANT-C. All of the studies that figure into our graphical meta-analysis are listed and categorized in Table 2. Some of these, such as Rueda et al. (2004), are developmental studies. Our categorization as “*developmental*” is not about the question addressed by a study, that could have been about attention and socio-economics (e.g., Mezzacappa 2004) or about attention and bilingualism (e.g., Antón et al. 2014), but rather, and simply, whether the study included some variation in age. Some studies used the ANT-C while only testing one age group. Usually, this was to ask a specific question (such as to compare children with and without some disorder). From these studies, we only included the data from the control (typically developing) children. Some scholars used a modified ANT-C that incorporated one or both features from the ANT-I (e.g., auditory warning signals and/or uninformative peripheral cues), while preserving the ANT-C’s child-friendly engagingness. Other scholars eschewed the ANT-C’s engagingness and explored attention in children using the original ANT.

Two developmental studies deserve specific mention and are presented separately because of their methodological uniqueness. Mullane et al. (2014) conducted a well-designed developmental study, with children aged 7–12, that used a modified version of the adult ANT-I. As described above, their modified version (M-ANT-I) provides a second measure of executive control, based on the Simon effect. Giovannoli et al. (2021) used the ANTI-V (developed by Roca et al. 2011), with cars replacing arrows as the target and distractor stimuli. As can be seen in panels A–D of Figure 2, Figure 3 and Figure 4, there is a dearth of evidence about changes in network scores during adolescence. A second unique feature of Giovannoli et al.’s work is that they have filled this gap with a relatively well-powered study.

**Table 2:** Studies used in the analysis of children and young adults, Figure 2, Figure 3 and Figure 4.

Study	Ages	N	Notes
ANT-C (Age Varied)			
Rueda et al. (2004, E1)	6–9+	48	
Rueda et al. (2004, E2)	10, 27	24	
Mezzacappa (2004)	5–7	241	
Forns et al. (2014)	7.5–9.5	2703	
Aubry and Bourdin (2018, 2021)	12, 21	168	Intellectually gifted and normal children were combined.
Antón et al. (2014) ^a^	7.5–11.5	360	Mono- and bi-lingual participants were combined.
ANT-C (one age)			
Hames et al. (2016)	16	6	
Kapa and Colombo (2013)	9.6	79	
Kooistra et al. (2011)	9.1	38	
Park et al. (2019)	10	58	
Ridderinkhof et al. (2020)	13	51	
Walczak-Kozłowska et al. (2020)	5.6	31	
ANT-O (Age varied)			
Baijal et al. (2011)	13–15	76	
Rueda et al. (2004, E2)	10, 27	24	
ANT-O (one age)			
Kuc et al. (2020)	13.2	72	
Raji et al. (2020)	14	10	
Twilhaar et al. (2018)	13.3	33	
ANT-I ^b^ (Age varied)			
Abundis-Gutiérrez et al. (2014)	5–24	60	
Casagrande et al. (2021)	5–6	44	Children 3 and 4 years old were excluded due to high error rates.
Pozuelos et al. (2014)	6–12	227	Experiments 1 and 2 were combined.
M-ANT-I			
Mullane et al. (2014)	7–12	96	
ANTI-V			
Giovannoli et al. (2021)	10–19	182	Participants were divided into three groups of adolescents.

Note. ^a^ Using the ANT-C, Anton et al. made the spatial cues uninformative. The alerting and executive scores from this study are included in this category, the orienting scores are included with the ANT-I studies. ^b^ All used versions of the ANT-I that were child-friendly in different ways.

Using the ANTI-Vea (developed by Luna et al. 2018 and thoroughly described by Coll-Martín et al. 2023) and with a focus on the relative age effect in school athletes (a disproportionately high percentage of athletes in sport teams were born early in the selection year) Huertas et al. (2019) recruited 10- and 12-year-old male soccer players. Their non-representative recruitment strategy, with its focus on male athletes, precludes presenting their findings in Figure 2, Figure 3 and Figure 4.

All of the studies that are used in our graphical analysis of the development of attention networks are listed and categorized in Table 2. The panels in Figure 2, Figure 3 and Figure 4 correspond roughly to these categories. 

### 6.1. Alerting

Graphical analysis of the development of alertness scores is presented in Figure 2. In Rueda et al.’s (2004) Experiment 1, whose data are plotted with filled stars (here and in Figure 3 and Figure 4), there was no significant variation in alertness scores between children aged 6 to a little over 9. In their Experiment 2, however, whose data are plotted as unfilled stars (here and in panel B, and in the corresponding panels of Figure 3 and Figure 4), which compared 10- and 27-year-old participants, a developmental trend is suggested. Moreover, it is clear from all of the panels that, when the corpus of all relevant studies is considered, there is a robust decrease in alertness scores during development, a trend that is substantially more robust when the young adult data points are removed. Although it seems reasonable to assume that, by the age of twelve, alertness scores have nearly achieved the level seen in young adults (aged 21–27), the similarity of the two linear functions in panel E suggests the possibility of a small further decrease between childhood and young adulthood.

### 6.2. Orienting

Graphical analysis of the development of orienting scores is presented in Figure 3. The first thing to note about these data is that the trends in the individual panels are considerably weaker than those seen for alerting. Secondly, with the ANT-C and ANT-O, the weak trends are increasing scores within the children (15 and under), while the opposite weak trend (scores decrease with age) is seen with the uninformative cues used in the ANT-I and M-ANTI-I. With the 100% informative cues, the changes between childhood and young adulthood are in opposite directions in the pair of observations from Rueda et al. (ANT-C in panel A and ANT-O in panel B), which makes it difficult to make a firm conclusion about changes between late childhood and early adulthood. In contrast, when uninformative cues are used (panel C), there is strong evidence that orienting scores continue to decrease past adolescence (see blue line). This trend is strongly reinforced by the similarly decreasing scores during adolescence in the Giovannoli et al. (2021) study (panel E).

The trend of increasing orienting scores with the ANT during childhood could reflect an increase in the ability of children to use the 100% informative cues to allocate attention. In contrast, uninformative cues are thought to capture attention despite that they do not predict the target’s upcoming location. Indeed, because targets are equally likely to appear at either location when cues are uninformative, such cues are sometimes described as distractors that the observer should try to ignore. The decrease with age in orienting scores in studies using uninformative peripheral cues (panels C, D and E in Figure 3) could be due to improvements in this ability.

### 6.3. Executive Control

Graphical analysis of the development of executive control scores is presented in Figure 4. Here, it can be seen that, in childhood (from 6 to 15 years of age), there is a robust improvement (decrease) in measures of executive control (whether from the flanker effect, in panels A–E, or the Simon effect, in panel F). Moreover, by the end of childhood, executive function scores from the flanker effect have almost achieved the level seen in young adults. Evidence from Abundis-Gutiérrez et al. (2014) (see highlighted data points, plus signs, from their 10.8- and 23.6-year-old participants in panel C) when combined with the adolescent data from Giovannoli et al.’s study (panel E) suggests continued improvement during childhood and adolescence, until the young -adult level of performance is obtained.

Because we have access to the raw data from Mullane, we can report that the correlation between the executive scores based on the flanker and Simon effects, while small r(94) = 0.26, is nevertheless significant (*p* = .01)^3^. The raw data from this study also allow us to explore the suggestion, at the end of the orienting section, that reductions in orienting scores as children get older might be due to improvements in executive control. In contrast to this suggestion, the correlations between the orienting scores and Mullane’s two measures of executive control (flanker and Simon) were near zero (0.04 and 0.00, respectively).

### 6.4. Summary and Interpretation

Alerting network scores generally decrease during childhood, whether the warning signals are visual or auditory. Developmental trends in either the level of tonic alertness or the ability to voluntarily prepare to respond to targets could generate such a pattern. If the tonic level of alertness were low in young children and increased during development then, with more room for improvement, warning signals would have a greater beneficial effect in younger children. Support for this possibility comes from Giovannoli et al. (2021), whose correlational analysis revealed that low levels of vigilance (which might reflect low levels of tonic alertness) were associated with higher alertness scores. Alternatively, typical warning signals (like those used in all the versions of the ANT) are thought to exert both exogenous (automatic) and endogenous (volitional) control over readiness to respond (cf. Lawrence and Klein 2013; Weinbach and Henik 2012). It seems likely that the endogenous component (ability to voluntarily use the warning signals to prepare for processing and responding to targets) improves with age. While the ubiquitous finding that reaction times are slower for younger children is consistent with a developmental improvement in tonic alertness, a developmental improvement in volitional control is not ruled out by this possibility. And, it should be noted that these two explanations are certainly not mutually exclusive. Alerting is generated by visual cues in the ANT-C and ANT-O, and by auditory signals in the ANT-I and M-ANT-I. After reviewing converging evidence from several different paradigms, Posner et al. (1976) concluded that “visual stimuli when unattended are less likely to alert the subject than stimuli occurring in other modalities” (p. 164). The similarity across modality of the signals used to generate alertness in these studies suggests that the visual signals were sufficiently attended to generate alertness in children.

The patterns with orienting are less robust. During childhood, there appears to be a trend for increasing orienting scores with 100% informative cues, and for decreasing scores with uninformative cues as the children age. The difference in the information value of these two types of cues could very well explain these developmental trends, i.e., younger children are less able to utilize information, while older children are better able to ignore (and resist capture by) uninformative cues.

The developmental pattern with the executive control network scores is robust, with a consistent improvement with age in the ability to ignore irrelevant flankers and/or to suppress any response conflict they might generate.

Using the ANT-O and with a focus on cortical thickness and its relation to attention, Boen et al. (2021) densely sampled in the age range from 8.5 to 27. Their analyses of their ANT data used ratio scores (rather than the unaltered subtractions as described in our Table 1) and we were unable to secure their data in a form that would permit inclusion in our graphic-meta-analyses. Nevertheless, careful inspection of the reaction time results presented in Figure 3 of their supplement allows us to offer the following descriptions: From age 8.5 to about 18 alerting scores decrease (from about 75 ms to about 40 ms) after which they are relatively stable; Despite an apparent decrease between 15 and 18 orienting scores are relatively stable (ranging between 60 ms and 45 ms) across the ages tested; From age 8.5 to about 18 executive scores decrease rapidly (from about 195 ms to about 90 ms) after which they are very stable. The patterns with alerting and executive control are very similar to what we report in Figure 2 and Figure 4.

## 7. Measuring the Networks of Attention during Adulthood

All of the studies used in our graphical meta-analysis of the effects of aging upon attention networks are listed and categorized in Table 3. Most of these studies (whose network scores are presented in panels A of Figure 4, Figure 5 and Figure 6) used the ANT-O, and most of these compared two or, in a few cases, three groups of participants with one group of young adults and one (or two) groups of older adults. One recent study (Veríssimo et al. 2022), which also used the ANT-O, did not include any adults under 58 years of age, but densely sampled older adults (58+). Two studies used unique versions of the ANT that are described next, as well as in the notes of Table 3.

Two published studies (Erel et al. 2020; Zivony et al. 2020) with different foci (alertness and orienting, respectively) relied upon the same data set that had been collected from six groups, with young and old participants experiencing three different versions of the ANT-O. For present purposes, we secured data from the authors,^4^ allowing us to generate the three network scores that are represented in panels B of Figure 4, Figure 5 and Figure 6. Two versions were very similar to the ANT-O, except for the probability of a target appearing at the location of the spatial cue. In one version, these cues were uninformative, as in the ANT-I (exogenous); in another version, these cues were 75% valid (endogenous, visual), as in the ANT-R (cf. Almeida et al. 2021, for a description). In the third version, the peripheral cues from the second version were replaced with spoken verbal cues (or no cue), “up”, “down” or both simultaneously, and the single-word cues were 75% predictive of the target’s location (endogenous, auditory).

**Table 3:** Studies used in the analysis of adults, Figure 5, Figure 6 and Figure 7.

Study	Ages	N	Notes
ANT-O (two or three age groups)			
Dash et al. (2019)	33, 75	38	Event-related fMRI with jittered cue-target SOAs.
Gamboz et al. (2010)	26, 68	135	
Jennings et al. (2007)	19, 69	123	
Kaufman et al. (2016)	23, 65	35	
Knight and Mather (2013)	21, 73	59	Morning and afternoon results were combined.
Mahoney et al. (2012)	19, 76	36	Data from six randomly intermixed uni- and multi-sensory cue conditions were combined.
Sperduti et al. (2016)	27, 67	35	Effect of meditation was excluded.
Williams et al. (2017)	22, 65	49	Gain/loss conditions were combined.
Young-Bernier et al. (2015)	22, 70	64	
Zhou et al. (2011)	28, 51, 71	90	
ANT-O (only old)			
Veríssimo et al. (2022)	58–93	643	Densely sampled this age range.
Other (two or three age groups)			
Casagrande et al. (2021) ^a^	23, 55, 71	171	Data from targets presented in the left and right hemifields were combined.
Lopez-Ramon et al. (2011)	31.5, 52.5	55	Used the ANT-I with a focus on error-proneness in drivers.
Erel/Zivony et al. (2020) ^b^	22, 72	238	Two publications, each with a different focus, were based on the same data.

Note. ^a^ Using the ANT-C, Anton et al. made the spatial cues uninformative. The alerting and executive scores from this study are included in this category, the orienting scores are included with the ANT-I studies. ^b^ All used versions of the ANT-I that were child-friendly in different ways.

Casagrande et al. (2021) tested three age groups, using a modified version of the lateralized ANT-I in which, instead of arrows pointing left and right, the targets were two different fruits (hence, LANTI-f). As noted in Table 3, for present purposes, we have collapsed the lateralized (left and right hemifield) network scores that were reported at the bottom of Figure 4 from this paper.^5^ Lopez-Ramon et al. (2011) used the standard ANT-I.

Some studies in our aging analysis used transformed RT to generate network scores (e.g., Erel/Zivony analysed z-transformed RT; Veríssimo et al. 2022 focussed on log(RT); Dash et al. 2019 used ratio scores). To permit us to compare studies on a level playing field, and because we believe that such transformations may conceal and/or generate differences not present in untransformed RTs, we obtained, from the papers, the authors, and/or on-line repositories, untransformed network scores from these studies. In the figures representing their findings, Veríssimo et al. presented network scores based on log(RT) as a continuous function of age, which was possible because of their large number of participants and dense sampling of older adults. For present purposes, we used their on-line repository (and a >90% correct cutoff per participant) to generate seven groups of participants (each with 80 or more participants), and we plotted their network scores (based on untransformed RTs) as a function of these age groups in panels D of Figure 5, Figure 6 and Figure 7.

### 7.1. Alerting

Graphical analysis of the effects of aging upon alertness scores is presented in Figure 5. In the studies using the ANT-O (panel A) with groups that included young adults, there is a robust effect of aging, with alerting scores decreasing as age increases, as can be seen in panel A. Of the studies represented in this panel, all 10 showed an effect in this direction (*p* = .00195, using a non-parametric two-tailed sign test). There is reinforcement of this robust pattern from the two conditions in the Erel/Zivony study (panel B) that used visual warning signals. The trend is in the opposite direction in the study by Casagrande et al. (2021), whose L-ANTI-F uses auditory warning signals (panel C). This opposite trend would seem to be supported by the pattern with auditory cues in Erel/Zivony. Although in neither study was the trend for increasing alertness scores with increasing age significant, the pattern is nevertheless different from that with visual warning signals. By comparing the alertness scores from Veríssimo et al.’s study, in which only older participants were tested (panel D), to those in the other three panels, it can be seen that this study is definitely anomalous, in that the mean values in this study fluctuate around zero, while the mean alertness scores from older participants in the remaining studies vary between 5 ms and 90 ms. Reinforcing this point, Mahoney et al. (2010) who tested 100 participants between 70 and 93 reported that their average alerting score was 23.5 ms.

### 7.2. Orienting

Graphical analysis of the effects of aging upon orienting scores are presented in Figure 6. In the studies using the ANT-O with groups that included young adults, there is a trend for orienting scores to increase with age, as can be seen in panel A. Here, 8 out of 10 studies reported differences in this direction.^6^ Reinforcing this trend, data from the informative visual cue condition, used by Erel/Zivony (panel B), were in the same direction. Amongst their large group of older participants (58 and older), Veríssimo et al. also found an increasing orienting network score (panel D). There are two reasons, however, to question this trend, even despite the very large number of participants in this study. Firstly, while their orienting network scores are all greater than zero, they are very low relative to what is seen in older participants in all of the other experiments (panels A–C). Secondly, consider the very robust (R^2^ = 0.4027) trend as a function of age (in the range from 65 to 87) in the relatively methodologically homogenous studies reported in panel A (see best fitting linear function, red line). 

### 7.3. Executive Control

Graphical analysis of the effects of aging upon executive control scores are presented in Figure 7. In the studies using the ANT-O with groups that included young adults, there is a robust effect of aging, with executive control scores increasing as age increases, as can be seen in Figure 7A. Of the 10 studies represented in this panel, 9 showed an effect in this direction (*p* = .0215, using a non-parametric, sign test).^7^ And, this pattern is reinforced by the three conditions explored by Erel/Zivony (panel B) and Casagrande et al. (panel C). Amongst the older participants tested by Veríssimo et al. (panel D), there was a small (from the youngest to oldest group, this was ~9 ms), but statistically robust, decrease in executive control scores with increasing age. This trend is reinforced by a similar (R^2^ = 0.1135) trend as a function of age (in the range from 65 to 87) in the relatively methodologically homogenous studies reported in panel A (see best fitting linear function, red line).

### 7.4. Summary and Interpretation

With visual warning signals, young adults show higher alertness scores than older adults, a difference that is not found when the warning signals are auditory. As noted earlier (see Section 7.1), the lower alertness scores in older participants with visual cues might be due to a difference in the amount of attention paid to them. That older participants do not show this difference when the warning signals are auditory, and may even show higher alertness scores with auditory signals, suggests that there is no global deficit in the alerting network of the older group.

When the orienting scores of young and older adults are compared, these are higher for the older participants in 9 out of 11 different experiments that used informative visual cues (cf. panels A and B of Figure 5). Amongst the older participants (aged 58 and above), there are opposite trends in the powerful study by Veríssimo et al. and the studies presented in Figure 5A. These trends noted, their significance is unclear. Higher scores could be due to more efficient allocation of attention to the cued location, or greater difficulty disengaging attention from the central cue (in the ANT-O) or a peripheral cue on invalid trials (in the ANT-I).

The effects of aging upon executive scores are relatively clearcut and consistent across the studies presented in Figure 7. When young adults and older adults are compared, executive scores are consistently higher for the older adults. Amongst older adults there is a consistent trend for executive scores to decrease as participants get older (Figure 7, panel A, red line; panel D).

## 8. General Discussion

In the preceding sections, we reviewed and graphically presented the findings from 37 publications to explore how the networks of attention, as described by Posner, might change across the lifespan. Before we try to describe and discuss the overall arcs of these changes in attention, a few comments are warranted. It is important for the reader to recognize that the graphical meta-analyses presented in our review are focussed on the patterns of the results generated in these 37 publications, and not on the descriptions or conclusions contained therein. As described in Fan and Posner (2004), it is also important to remember that only the magnitude of the executive control score has an unambiguous meaning when asserting improvement or deterioration in performance; higher executive scores (whether based on RT or error rates, using the subtraction described in Table 1) represent poorer performance (usually attributed to less efficient filtering of task-irrelevant information), and hence, less efficient executive control. Differences in the alerting and orienting network scores, while of interest if not importance, are somewhat ambiguous on the improvement/deterioration scale. Alerting scores can be higher because of more efficient use of the warning signal to prepare for the target or because the participant’s level of alertness is lower to start with. Similarly, orienting scores can be larger because of more efficient use of the cue when meaningful, because of greater capture of attention by uninformative cues (e.g., as in the ANT-I) and because of a greater difficulty disengaging from the central cue (in the ANT-O) or the invalidly cued location (in the ANT-I and versions of the ANT-O with invalid trials).

### 8.1. Summary: Attention Network Changes across the Lifespan

Based on our analysis of network changes in development and aging, with an emphasis on reaction time, it is possible to draw the following lifespan descriptions with some confidence.

When visual warning signals are used, alerting network scores decrease monotonically from young childhood to old age. A different pattern emerges with auditory cues. Here, while the decline during childhood is robust, the few studies using auditory warnings to examine aging observed an increase with increasing age.

The results with orienting seem to depend on whether the cues are or are not informative. With uninformative cues, exogenous orienting scores decrease during childhood, reach a minimum during young adulthood, and then increase during aging. When informative cues are used, the picture from the literature we have reviewed is a bit messy. During childhood, endogenous orienting scores decrease, but whether these scores increase or decrease between childhood and young adulthood is unclear. Finally, there is a trend for orienting scores to increase from young adulthood with increasing age.

The executive control scores are high for young children, decrease during childhood, reach an optimal level during young adulthood, and then increase during aging. Converging evidence for this U-shaped function is provided by the flanker effect in RT, observed by Erb et al. (2023) in their lifespan comparison of the flanker, Stroop, and Simon effects. It is possible, however, that during old age, executive control scores may improve (decrease) with age. 

The relatively consistent increase in executive network scores as age increases during adulthood, as seen in Figure 7A, which represents a decline in filtering efficiency, is qualified by an opposite trend in error rate, such that young adults show larger flanker compatibility effects than older adults.^8^ This pattern was first reported by D’Aloisio and Klein (1990), whose flanker effect was measured as a function of distance between equi-eccentric targets and flankers. Indeed, when taking both RT and accuracy into account, they concluded that the effect of flankers, as a function of their distance from the target, was similar for their groups of older and younger participants. Most studies we analysed in this review did not report data about errors or accuracy. However, in all of the studies that did report (or provided to us) accuracy or error rates, older participants had lower executive function scores than younger participants when scores were based on error rates. This means that some, indeterminate, amount of the increase in executive network scores with aging, based on RT, is due to a speed–accuracy trade-off, wherein the younger participants show smaller effects in RT by caring less about errors than the older participants, reflecting a general and well-known speed–accuracy emphasis difference between these age groups (cf. Ratcliff et al. 2004).

### 8.2. Relation to Intelligence?

Two of the studies included in our graphical meta-analysis (Casagrande et al. 2021; Mullane et al. 2014) collected data about the intelligence of their participants, and the authors of these studies generously provided us with these scores. Particularly because this review is submitted to a Special Issue of the *Journal of Intelligence*, it seems appropriate to report on what we have found about the possible relation between IQ and the networks of attention. As can be seen in Table 4, there is only one significant correlation, a negative one, between orienting and IQ amongst the adult participants. While this correlation is relatively weak, at −0.172, the true correlation could be larger because the observed correlation is likely attenuated by non-perfect reliability of the IQ and orienting scores. The trend (albeit non significant) in the opposite direction (+0.138), observed in the developmental study, could be regarded as a reason to imagine that the true correlation is nil. On the other hand, it is worth considering whether this directional difference in the relation between IQ and orienting as a function of age simply reflects and reinforces our description of the lifespan trend for orienting scores observed in studies with uninformative cues (as in the two studies represented in Table 4). With such cues, “…orienting scores decrease during childhood, reach a minimum during young adulthood, and then increase during aging”.

**Table 4:** Correlations between IQ and network scores from two studies (one developmental; one aging) covered in our review. Correlations significantly greater than zero are highlighted in bold.

Study	Age Range	N	Alerting	Orienting	Executive Control
(Mullane et al. 2014)	79–150 months	96	−0.060	0.138	−0.143
(Casagrande et al. 2021)	20–86 years	171	0.053	**−0.172**	−0.050

After a cogent and thorough review and analysis, Mashburn et al. (2023) recently concluded that “executive attention” is fundamental for “explaining variation in human intelligence”. If this conclusion were correct, we might have expected significantly negative correlations between IQ and the executive scores measured using the flanker effects in the two studies represented in Table 4. In agreement with Mashburn et al., these correlations are negative, but they are far from significant. There are a few possible explanations for this apparent failure to confirm the prediction we derived from Mashburn et al., and these are not mutually exclusive. For example, the flanker effect may measure something about executive control, but executive attention may be considerably more complex than what can be measured by this simple subtraction (incongruent minus congruent). Mashburn et al. provide another possibility, which is related to the use of such a difference score: because of their generally low reliability, “difference scores derived from conflict tasks are ill-suited for individual differences research (p. 15)”. But, the low reliability of difference scores does not similarly challenge comparisons of groups of participants (cf. Hedge et al. 2018). Indeed, supporting Mashburn et al., Aubry and Bourdin (2021) compared intellectually gifted (mean IQ = 140) to intellectually normal children (mean IQ = 103) and found a significant executive control advantage (as would be predicted from Mashburn et al.’s aforementioned conclusion) in the group with the higher IQ.

### 8.3. Next Steps

As noted in the introduction, while there are a few lifespan studies of individual aspects of attention, there are no lifespan studies using tests, like the ANT, that measure all three of the networks outlined by Posner and colleagues. This gap could be filled by researchers using a version of the ANT that is engaging for both children and adults, while densely and relatively uniformly sampling ages. One possibility for such a lifespan study would be the ANT-C, developed by Rueda et al. (2004) specifically for use with children, but also successfully administered in their Experiment 2 to young adults (see panels A in Figure 2, Figure 3 and Figure 4). Another possibility for such a lifespan study would be to use the Attention-Trip (Klein et al. 2017), a game-like version of the ANT. The Attention-Trip has been shown to be engaging for children (Arora et al. 2021) and was recently made operational on a portable device, the iPad (Klein et al. 2018). 

## Data Availability

Not applicable.

## Notes

1 We are grateful to Jennifer Mullane for providing access to the data from her individual participants.

2 Because this is the only study covered in our review with a measure of vigilance, we refer the reader interested in the development of vigilance to Giovannoli et al. (2021).

3 This is in contrast to the null correlation reported by Paap and Greenberg (2013) with an adult sample, a difference that might be related to the ages of participants.

4 We are grateful to Alon Zivony for providing us with the mean RT and accuracy data from the the individual participants in the Erel/Zivony publications.

5 Although not required for the data presented in Figure 4, Figure 5 and Figure 6, we are grateful to Maria Casagrande for providing the RT and accuracy data for the individual participants in her study, which will be used later for other purposes.

6 Dash et al. (2019) and Mahoney et al. (2012) were the two exceptions. Dash et al. administered the ANT-O using an event-related fMRI protocol, in which the interval between the onset of the cue and target array was varied, between 600 and 6600 ms, which makes it quite different from all of the other studies in our aging analysis. Perhaps the older participants had difficulty maintaining the focus of attention at the cued location when the cue-target SOA was so long. Mahoney et al. used uni- and multi-sensory alerting+orienting cues (visual, auditory, and somatosensory). It seems possible that orienting within the visual modality may have been compromised in the older participants by the spreading of attention amongst the three modalities in which the cues might be presented on any particular trial.

7 The single exception was Dash et al. (2019), whose old and young participants had executive scores of 46.6 and 72.3 ms, respectively. Although this study was unusual with regard to the range of foreperiods between the cue and target (see footnote #5) it is difficult to generate a plausible reason for this to matter for executive control.

8 Erb et al. (2023) collected flanker compatibility effects from a very large sample of on-line participants who experienced congruent and incongruent target arrays modelled on those used in the ANT. In contrast to what is described here, they found that accuracy flanker effects increased with age from young to older adults. We can offer no explanation for this discrepancy.

## References

[B1-jintelligence-12-00019] Abundis-Gutiérrez Alicia, Checa Purificación, Castellanos Concepción, Rueda M. R. (2014). Electrophysiological correlates of attention networks in childhood and early adulthood. Neuropsychologia.

[B2-jintelligence-12-00019] Almeida Rafael, Faria Aydamari, Klein Raymond M. (2021). On the origins and evolution of the Attention Network Tests. Neuroscience & Biobehavioral Reviews.

[B3-jintelligence-12-00019] Antón Eneko, Duñabeitia Jon A., Estévez Adelina, Hernández Juan A., Castillo Alejandro, Fuentes Luis J., Davidson Douglas J., Carreiras Manuel (2014). Is there a bilingual advantage in the ANT task? Evidence from children. Frontiers in Psychology.

[B4-jintelligence-12-00019] Arora Swasti, McCormick Colin R., Klein Raymond M. (2021). Comparing Youth Engagement on the AttentionTrip to the Child Attention Network Test. International Journal of Human–Computer Interaction.

[B5-jintelligence-12-00019] Arora Swasti, Lawrence Michael A., Klein Raymond M. (2020). The Attention Network Test Database and Some Examples of its Application. Frontiers in Psychology.

[B6-jintelligence-12-00019] Aubry Alexandre, Bourdin Béatrice (2018). Development of attentional networks in intellectually gifted children. OSF.

[B7-jintelligence-12-00019] Aubry Alexandre, Bourdin Béatrice (2021). Alerting, orienting, and executive control in intellectually gifted children. Brain and Behavior.

[B8-jintelligence-12-00019] Baijal Shruti, Jha Amishi, Kiyonaga Anastasia, Singh Richa, Srinivasan Narayanan (2011). The influence of concentrative meditation training on the development of attention networks during early adolescence. Frontiers in Psychology.

[B9-jintelligence-12-00019] Baltes Paul B., Reese Hayne W., Lipsitt Lewis P. (1980). Life-span developmental psychology. Annual Review of Psychology.

[B10-jintelligence-12-00019] Berger Andrea, Jones Laura, Rothbart Mary K., Posner Michael I. (2000). Computerized games to study the development of attention in childhood. Behavioral Research Methods and Instrumentation.

[B11-jintelligence-12-00019] Boen Rune, Ferschmann Lia, Vijayakumar Nandita, Overbye Knut, Fjell Anders M., Espeseth Thomas, Tamnes Christian K. (2021). Development of attention networks from childhood to young adulthood: A study of performance, intraindividual variability and cortical thickness. Cortex.

[B12-jintelligence-12-00019] Broadbent Donald E. (1958). Perception and Communication.

[B13-jintelligence-12-00019] Callejas Alicia, Lupiánez Juan, Tudela Pıo (2004). The three attentional networks: On their independence and interactions. Brain and Cognition.

[B14-jintelligence-12-00019] Casagrande Maria, Agostini Francesca, Favieri Francesca, Forte Giuseppe, Giovannoli Jasmine, Guarino Angela, Marotta Andrea, Doricchi Fabrizio, Martella Diana (2021). Age-related changes in hemispherical specialization for attentional networks. Brain Sciences.

[B15-jintelligence-12-00019] Chomsky Noam (1959). Review of Verbal Behavior, by B. F. Skinner. Language.

[B16-jintelligence-12-00019] Coll-Martín Tao, Román-Caballero Rafael, Martínez-Caballero María Del Rocío, Martín-Sánchez Paulina Del Carmen, Trujillo Laura, Cásedas Luis, Castellanos M Concepción, Hemmerich Klara, Manini Greta, Aguirre María Julieta (2023). The ANTI-Vea-UGR platform: A free online resource to measure attentional networks (alertness, orienting, and executive control) functioning and executive/arousal vigilance. Journal of Intelligence.

[B17-jintelligence-12-00019] Craik Kenneth J. W. (1943). The Nature of Explanation.

[B18-jintelligence-12-00019] D’Aloisio April, Klein Raymond M., Enns J. (1990). Aging and the deployment of visual attention. The Development of Attention: Research and Theory.

[B19-jintelligence-12-00019] Dash Tanya, Berroir Pierre, Joanette Yves, Ansaldo Ana I. (2019). Alerting, orienting, and executive control: The effect of bilingualism and age on the subcomponents of attention. Frontiers in Neurology.

[B20-jintelligence-12-00019] Donders Franciscus C. (1969). On the speed of mental processes. Acta Psychologica.

[B21-jintelligence-12-00019] Enns James T. (1990). The Development of Attention: Research and Theory.

[B22-jintelligence-12-00019] Erb Christopher D., Germine Laura, Hartshorne Joshua K. (2023). Cognitive control across the lifespan: Congruency effects reveal divergent developmental trajectories. Journal of Experimental Psychology: General.

[B23-jintelligence-12-00019] Erel Hadas, Zivony Alon, Levy Daniel A. (2020). Cognitive processes in aging effects on attentional alerting. Neurobiology of aging.

[B24-jintelligence-12-00019] Eriksen Barbara A., Eriksen Charles W. (1974). Effects of noise letters upon the identification of a target letter in a nonsearch task. Perception & Psychophysics.

[B25-jintelligence-12-00019] Fan Jin, Posner Michael (2004). Human attentional networks. Psychiatr Praxis.

[B26-jintelligence-12-00019] Fan Jin, McCandliss Bruce D., Sommer Tobias, Raz Amir, Posner Michael I. (2002). Testing the efficiency and independence of attentional networks. Journal of Cognitive Neuroscience.

[B27-jintelligence-12-00019] Forns Joan, Esnaola Mikel, López-Vicente Mónica, Suades-González Elisabet, Alvarez-Pedrerol Mar, Julvez Jordi, Grellier James, Sebastián-Gallés Núria, Sunyer Jordi (2014). The n-back Test and the Attentional Network Task as measures of child neuropsychological development in epidemiological studies. Neuropsychology.

[B28-jintelligence-12-00019] Fortenbaugh Francesca C., DeGutis Joseph, Germine Laura, Wilmer Jeremy B., Grosso Mallory, Russo Kathryn, Esterman Michael (2015). Sustained attention across the life span in a sample of 10,000: Dissociating ability and strategy. Psychological Science.

[B29-jintelligence-12-00019] Gamboz Nadia, Zamarian Stefania, Cavallero Corrado (2010). Age-related differences in the attention network test (ANT). Experimental Aging Research.

[B30-jintelligence-12-00019] Giovannoli Jasmine, Martella Diana, Casagrande Maria (2021). Assessing the three attentional networks and vigilance in the adolescence stages. Brain Sciences.

[B31-jintelligence-12-00019] Hames Elizabeth C., Rajmohan Ravi, Fang Dan, Anderson Ronald, Baker Mary, Richman David M., O’Boyle Michael (2016). Attentional networks in adolescents with high-functioning autism: An fMRI investigation. Open Neuroimaging Journal.

[B32-jintelligence-12-00019] Hebb Donald O. (1949). The Organization of Behaviour: A Neuropsychological Theory.

[B33-jintelligence-12-00019] Hedge Craig, Powell Georgina, Sumner Petroc (2018). The reliability paradox: Why robust cognitive tasks do not produce reliable individual differences. Behavior Research Methods.

[B34-jintelligence-12-00019] Hommel Bernhard, Li Karen Z., Li Shu C. (2004). Visual search across the life span. Developmental Psychology.

[B35-jintelligence-12-00019] Huertas Florentino, Ballester Rafael, Gines Honorato José, Hamidi Abdel K., Moratal Consuelo, Lupiáñez Juan (2019). Relative Age Effect in the Sport Environment. Role of Physical Fitness and Cognitive Function in Youth Soccer Players. International Journal of Environmental Research and Public Health.

[B36-jintelligence-12-00019] James William (1890). The Principles of Psychology.

[B37-jintelligence-12-00019] Jennings Janine M., Dagenbach Dale, Engle Christine M., Funke Laura J. (2007). Age-related changes and the attention network task: An examination of alerting, orienting, and executive function. Aging, Neuropsychology, and Cognition.

[B38-jintelligence-12-00019] Kapa Leah L., Colombo John (2013). Attentional control in early and later bilingual children. Cognitive Development.

[B39-jintelligence-12-00019] Kaufman David A., Sozda Christopher N., Dotson Vonetta M., Perlstein William M. (2016). An event-related potential investigation of the effects of age on alerting, orienting, and executive function. Frontiers in Aging Neuroscience.

[B40-jintelligence-12-00019] Klein Raymond M. (2003). Chronometric explorations of disordered minds. Trends in Cognitive Sciences.

[B41-jintelligence-12-00019] Klein Raymond M. (2009). On the Control of Attention. Canadian Journal of Experimental Psychology.

[B42-jintelligence-12-00019] Klein Raymond M. (2022). Thinking about attention: Successive approximations to a productive taxonomy. Cognition.

[B43-jintelligence-12-00019] Klein Raymond M., Arora S., McCormick C., Rehan S. (2018). Testing the networks of attention: From the ANT to the AttentionTrip. Paper presented at the 59th Meeting of the Psychonomic Society.

[B44-jintelligence-12-00019] Klein Raymond M., Hassan Tariq, Wilson Graham, Ishigami Yoko, Mulle Jonathan (2017). The AttentionTrip: A game-like tool for measuring the networks of attention. Journal of Neuroscience Methods.

[B45-jintelligence-12-00019] Knight Marisa, Mather Mara (2013). Look out—It’s your off-peak time of day! Time of day matters more for alerting than for orienting or executive attention. Experimental Aging Research.

[B46-jintelligence-12-00019] Kooistra Libbe, Crawford Susan, Gibbard Ben, Kaplan Bonnie J., Fan Jin (2011). Comparing attentional networks in fetal alcohol spectrum disorder and the inattentive and combined subtypes of attention deficit hyperactivity disorder. Developmental Neuropsychology.

[B47-jintelligence-12-00019] Kuc Katarzyna, Bielecki Maksymilian, Racicka-Pawlukiewicz Ewa, Czerwinski Michał B., Cybulska-Klosowicz Anita (2020). The SLC6A3 gene polymorphism is related to the development of attentional functions but not to ADHD. Scientific Reports.

[B48-jintelligence-12-00019] Lachman Roy, Lachman Janet L., Butterfield Earl C. (1979). Cognitive Psychology and Information Processing: An Introduction.

[B49-jintelligence-12-00019] Lawrence Michael A., Klein Raymond M. (2013). Isolating exogenous and endogenous mechanisms of temporal attention. Journal of Experimental Psychology: General.

[B50-jintelligence-12-00019] Lopez-Ramon María F., Castro Cándida, Roca Javier, Ledesma Rubén, Lupianez Juan (2011). Attentional Networks Functioning, Age, and Attentional Lapses While Driving. Traffic Injury Prevention.

[B51-jintelligence-12-00019] Luna Fernando G., Marino Julián, Roca Javier, Lupiáñez Juan (2018). Executive and arousal vigilance decrement in the context of the attentional networks: The ANTI-Vea task. Journal of Neuroscience Methods.

[B52-jintelligence-12-00019] Mahoney Jeannette R., Verghese Joe, Dumas Kristina, Wang Cuiling, Holtzer Roee (2012). The effect of multisensory cues on attention in aging. Brain Research.

[B53-jintelligence-12-00019] Mahoney Jeannette R., Verghese Joe, Goldin Yelena, Lipton Richard, Holtzer Roee (2010). Alerting, orienting, and executive attention in older adults. Journal of the International Neuropsychological Society.

[B54-jintelligence-12-00019] Mashburn Cody A., Barnett Mariel K., Engle Randall W. (2023). Processing Speed and Executive Attention as Causes of Intelligence. Psychological Review.

[B55-jintelligence-12-00019] McAvinue Laura P., Habekost Thomas, Johnson Katherine A., Kyllingsbæk Søren, Vangkilde Signe, Bundesen Claus, Robertson Ian H. (2012). Sustained attention, attentional selectivity, and attentional capacity across the lifespan. Attention, Perception, & Psychophysics.

[B56-jintelligence-12-00019] Mezzacappa Enrico (2004). Alerting, orienting, and executive attention: Developmental properties and sociodemographic correlates in an epidemiological sample of young, urban children. Child Development.

[B57-jintelligence-12-00019] Mullane Jennifer C., Lawrence Michael A., Corkum Penny V., Klein Raymond M., McLaughlin Elizabeth N. (2014). The development of and interaction among alerting, orienting, and executive attention in children. Child Neuropsychology.

[B58-jintelligence-12-00019] Paap Kenneth R., Greenberg Zachary I. (2013). There is no coherent evidence for a bilingual advantage in executive processing. Cognitive Psychology.

[B59-jintelligence-12-00019] Park Jissok, Miller Carol A., Sanjeevan Teenu, van Hell Janet G., Weiss Daniel J., Mainela-Arnold Elina (2019). Bilingualism and Attention in Typically Developing Children and Children With Developmental Language Disorder. Journal of Speech, Language, and Hearing Research.

[B60-jintelligence-12-00019] Plude Dana J., Enns Jim T., Brodeur Darlene (1994). The development of selective attention: A life-span overview. Acta Psychologica.

[B61-jintelligence-12-00019] Posner Michael I. (1978). Chronometric Explorations of Mind.

[B62-jintelligence-12-00019] Posner Michael I. (1980). Orienting of attention. Quarterly Journal of Experimental Psychology.

[B63-jintelligence-12-00019] Posner Michael I., Boies Stephen J. (1971). Components of attention. Psychological Review.

[B64-jintelligence-12-00019] Posner Michael I., Petersen Steven E. (1990). The attention system of the human brain. Annual Review of Neuroscience.

[B65-jintelligence-12-00019] Posner Michael I. (2016). The Psychology of Attention.

[B66-jintelligence-12-00019] Posner Michael I., Nissen Mary J., Klein Raymond M. (1976). Visual dominance: An information-processing account of its origins and significance. Psychological Review.

[B67-jintelligence-12-00019] Pozuelos Joan P., Paz-Alonso Pedro M., Castillo Alejandro, Fuentes Luis J., Rueda M Rosario (2014). Development of attention networks and their interactions in childhood. Developmental Psychology.

[B68-jintelligence-12-00019] Raji Cyrus A., Wang Maxwell B., Nguyen NhuNhu, Owen Julia P., Palacios Eva M., Yuh Esther L., Mukherjee Pratik (2020). Connectome mapping with edge density imaging differentiates pediatric mild traumatic brain injury from typically developing controls: Proof of concept. Pediatric Radiology.

[B69-jintelligence-12-00019] Ratcliff Roger, Thapar Anjali, Gomez Pablo, McKoon Gail (2004). A diffusion model analysis of the effects of aging in the lexical-decision task. Psychology and Aging.

[B70-jintelligence-12-00019] Ridderinkhof Anna, de Bruin Esther I., van den Driesschen Sanne, Bögels Susan M (2020). Attention in children with autism spectrum disorder and the effects of a mindfulness-based program. Journal of Attention Disorders.

[B71-jintelligence-12-00019] Roca Javier, Castro Cándida, López-Ramón María Fernanda, Lupiáñez Juan (2011). Measuring vigilance while assessing the functioning of the three attentional networks: The ANTI-Vigilance task. Journal of Neuroscience Methods.

[B72-jintelligence-12-00019] Rueda M. Rosario, Fan Jin, McCandliss Bruce D., Halparin Jessica D., Gruber Dana B., Lercari Lisha Pappert, Posner Michael I. (2004). Development of attentional networks in childhood. Neuropsychologia.

[B73-jintelligence-12-00019] Shannon Claude Elwood (1948). A mathematical theory of communication. The Bell System Technical Journal.

[B74-jintelligence-12-00019] Sperduti Marco, Makowski Dominique, Piolino Pascale (2016). The protective role of long-term meditation on the decline of the executive component of attention in aging: A preliminary cross-sectional study. Aging, Neuropsychology, and Cognition.

[B75-jintelligence-12-00019] Titchener Edward B. (1908). Lectures on the Elementary Psychology of Feeling and Attention.

[B76-jintelligence-12-00019] Tolman Edward C. (1948). Cognitive maps in rats and men. Psychological Review.

[B77-jintelligence-12-00019] Trick Lana M., Enns James T. (1998). Lifespan changes in attention: The visual search task. Cognitive Development.

[B78-jintelligence-12-00019] Twilhaar E. Sabrina, de Kieviet Jorrit F., Oosterlaan Jaap, van Elburg Ruurd M. (2018). A randomised trial of enteral glutamine supplementation for very preterm children showed no beneficial or adverse long-term neurodevelopmental outcomes. Acta Paediatrica.

[B79-jintelligence-12-00019] Veríssimo João, Verhaeghen Paul, Goldman Noreen, Weinstein Maxine, Ullman Michael T. (2022). Evidence that ageing yields improvements as well as declines across attention and executive functions. Nature Human Behaviour.

[B80-jintelligence-12-00019] Walczak-Kozłowska Tamara, Mańkowska Aleksandra, Chrzan-Dętkoś Magdalena, Harciarek Michał (2020). Attentional system of very prematurely born preschoolers. Developmental Psychology.

[B81-jintelligence-12-00019] Waszak Florian, Li Shu-Chen, Hommel Bernhard (2010). The development of attentional networks: Cross-sectional findings from a life span sample. Developmental Psychology.

[B82-jintelligence-12-00019] Watson John B. (1913). Psychology as the behaviorist views it. Psychological Review.

[B83-jintelligence-12-00019] Weinbach Noam, Henik Avishai (2012). Temporal orienting and alerting—The same or different?. Frontiers in Psychology.

[B84-jintelligence-12-00019] Williams Ryan S., Biel Anna Lena, Dyson Benjamin J., Spaniol Julia (2017). Age differences in gain-and loss-motivated attention. Brain and Cognition.

[B85-jintelligence-12-00019] Young-Bernier Marielle, Tanguay Annick N., Tremblay François, Davidson Patrick S. R. (2015). Age Differences in Reaction Times and a Neurophysiological Marker of Cholinergic Activity. Canadian Journal on Aging/La Revue Canadienne du Vieillissement.

[B86-jintelligence-12-00019] Zelazo Philip David, Craik Fergus I. M., Booth Laura (2004). Executive function across the life span. Acta Psychologica.

[B87-jintelligence-12-00019] Zhou Shan-Shan, Fan Jin, Lee Tatia M. C., Wang Chang-Qing, Wang Kai (2011). Age-related differences in attentional networks of alerting and executive control in young, middle-aged, and older Chinese adults. Brain and Cognition.

[B88-jintelligence-12-00019] Zivony Hadas Erel, Levy Daniel A. (2020). Predictivity and manifestation factors in aging effects on the orienting of spatial attention. The Journals of Gerontology: Series B Psychological Sciences and Social Sciences.

